# Synaptic Activation of a Detailed Purkinje Cell Model Predicts Voltage-Dependent Control of Burst-Pause Responses in Active Dendrites

**DOI:** 10.3389/fncel.2017.00278

**Published:** 2017-09-13

**Authors:** Stefano Masoli, Egidio D’Angelo

**Affiliations:** ^1^Department of Brain and Behavioral Sciences, University of Pavia Pavia, Italy; ^2^Brain Connectivity Center, C. Mondino National Neurological Institute Pavia, Italy

**Keywords:** Purkinje cell, cerebellum, modeling, synapses, dendrites

## Abstract

The dendritic processing in cerebellar Purkinje cells (PCs), which integrate synaptic inputs coming from hundreds of thousands granule cells and molecular layer interneurons, is still unclear. Here we have tested a leading hypothesis maintaining that the significant PC output code is represented by burst-pause responses (BPRs), by simulating PC responses in a biophysically detailed model that allowed to systematically explore a broad range of input patterns. BPRs were generated by input bursts and were more prominent in Zebrin positive than Zebrin negative (Z+ and Z−) PCs. Different combinations of parallel fiber and molecular layer interneuron synapses explained type I, II and III responses observed *in vivo*. BPRs were generated intrinsically by Ca-dependent K channel activation in the somato-dendritic compartment and the pause was reinforced by molecular layer interneuron inhibition. BPRs faithfully reported the duration and intensity of synaptic inputs, such that synaptic conductance tuned the number of spikes and release probability tuned their regularity in the millisecond range. Interestingly, the burst and pause of BPRs depended on the stimulated dendritic zone reflecting the different input conductance and local engagement of voltage-dependent channels. Multiple local inputs combined their actions generating complex spatio-temporal patterns of dendritic activity and BPRs. Thus, local control of intrinsic dendritic mechanisms by synaptic inputs emerges as a fundamental PC property in activity regimens characterized by bursting inputs from granular and molecular layer neurons.

## Introduction

Purkinje cells (PCs) are the final common collector of the whole neuronal activity generated in the granular and molecular layer of the cerebellar cortex (Eccles et al., 1967; Ramón y Cajal, 1995). A complex structural and functional organization allows PCs to elaborate the largest synaptic input of the brain, amounting to over 200,000 synapses in rodents (Korbo et al., 1993). PCs are characterized by active dendrites (Llinás and Sugimori, 1980a,b) endowed with different types of voltage-dependent channels. Several experiments and models have been developed with the aim to explain how PCs integrate synaptic inputs (De Schutter and Bower, 1994a,b; Rapp et al., 1994; Roth and Häusser, 2001; Brunel et al., 2004; Santamaria and Bower, 2005; Steuber et al., 2007; Masoli et al., 2015), but the role of voltage-dependent currents in local computation has not been fully clarified. PCs have recently been proposed to react to input bursts by generating a burst-pause response (BPR), which has been correlated with animal behavior (Cao et al., 2012; Herzfeld et al., 2015). While the intervention of inhibitory molecular layer interneurons (molecular layer interneurons; Barmack and Yakhnitsa, 2008; Bower, 2010; Grasselli et al., 2016) has been proposed to regulate the pause, the role played by dendritic properties in BPR generation remained unclear.

BPRs are thought to reflect a stereotyped PC response to bursting granular layer inputs. The granule cells generate brief spike bursts *in vitro* (D’Angelo et al., 1995; Nieus et al., 2006) and *in vivo* (Chadderton et al., 2004; van Beugen et al., 2013; Powell et al., 2015) in response to bursts in the mossy fibers (Rancz et al., 2007). These granule cell bursts are finely regulated by GoC inhibition (Mapelli et al., 2009; Nieus et al., 2014) generating specific patterns in the number and timing of spikes (D’Angelo and De Zeeuw, 2009; Arleo et al., 2010). The granule cell bursts are then conveyed to molecular layer activating PCs and molecular layer interneurons, which in turn generate feed-forward inhibition on PCs (Santamaria et al., 2007; Rieubland et al., 2014; Zhang and Südhof, 2016). Therefore, granule cells and molecular layer interneuron set-up complex spatio-temporal patterns of activity generating PC responses that have been classified as type I, II and III (De Zeeuw et al., 2011) and are differentiated depending on Zebrin positive or negative (Z+ or Z−) PCs in different cerebellar areas (Zhou et al., 2014, 2015).

Since synaptic activity patterns impinging on PCs are not fully known and since monitoring the PC dendritic response is challenging, a first insight into the way PCs respond to their inputs can be obtained using *in silico* simulations. Here we have faced the issue by exploiting a detailed PC model (Masoli et al., 2015) that was extended with excitatory and inhibitory synapses. This model is auto-rhythmic and its electroresponsiveness has been validated against a large set of biological experiments providing an ideal substrate to explore how intrinsic electroresponsiveness is modulated by synaptic inputs. Simulations with this PC model allowed us to face a set of questions. Does the PC model generate BPR in response to synaptic inputs? Is BPR different in Z+ and Z− PC models? Is BPR affected by the specific location of inputs on dendritic branches, as in the case of ascending axons (aa) vs. parallel fiber inputs (Sims and Hartell, 2005, 2006; Walter et al., 2009)? Is BPR different when inputs are randomly distributed rather than concentrated in limited sub-regions? What is the impact on BPR of the number of spikes and of the intensity of inhibition in the input burst? What are the mechanisms of BPR (intrinsic electroresponsiveness or synaptic inhibition) and what is the role of voltage-dependent ionic channels? Can BPR be modulated by synaptic plasticity? Simulations can provide a coherent mechanistic hypothesis on this broad range of questions that would be otherwise be hard to achieve.

The simulations showed that, for a broad set of activity regimens, PCs generate BPRs modulated by the excitatory/inhibitory balance and by synaptic plasticity. The underlying BPR mechanism reflects modulation of intrinsic pacemaking by Ca/KCa currents generated in the dendritic compartment and transmitted to the soma and axon initial segment (AIS) through the internal resistance. Therefore, BPR emerges as a relevant coding strategy for PCs that could be modulated by spatio-temporal granule cell spike patterns and synaptic inhibition and plasticity, generating the specific outputs to be transmitted to DCN.

## Materials and Methods

The present synaptic PC model is based on the recent PC model of intrinsic electroresponsiveness by Masoli et al. (2015), which in turn derives its morphology from Rapp et al. (1994). The present model updates in several respects the previous active model by De Schutter and Bower (1994a,b) by incorporating a completely new set of ionic channels, by dislocating the action potential generation mechanism in the AIS and axon, by using dynamic synapses and by exploiting a wide set or recent experimental evidences for validation. The model by Masoli et al. (2015) (available on ModelDB), which was built to reproduce PC responses to current injections *in vitro* and *in vivo*, is extended here by connecting excitatory and inhibitory synapses on the dendrites and by evaluating a large set of combinations in the input space. As a further update to account for recent *in vivo* data, the maximum conductance of some ionic channels (soma HCN1 = 0.001 mS/cm^2^; soma Kv3.4 = 0.0515 mS/cm^2^; AIS Nav1.6 = 0.8 mS/cm^2^) was adjusted to raise the average frequency from 35 Hz to 40 Hz in the Z+ PC model (Zhou et al., 2014). The Z− differed Z+ models only for the presence of TRPC channels. TRPC channels are cationic channels generating a tonic depolarizing current, which, once placed in Z− PCs, raised background frequency to around 90 Hz (dendrite TRPC = 4.18e^−6^ mS/cm^2^). All simulations were performed at 37° with fixed time step (0.025 ms) and were run using NEURON multisplit (Python 2.7; NEURON 7.5; Hines et al., 2007, 2009) to exploit the eight-core processor of an AMD FX 8350 with 16 GB RAM and an AMD Ryzen 1800x 8 cores/16 threads with 32 GB RAM. The data were analyzed with custom python and MATLAB scripts.

### Synaptic Modeling

#### Synaptic Distribution

**Figure 1:**
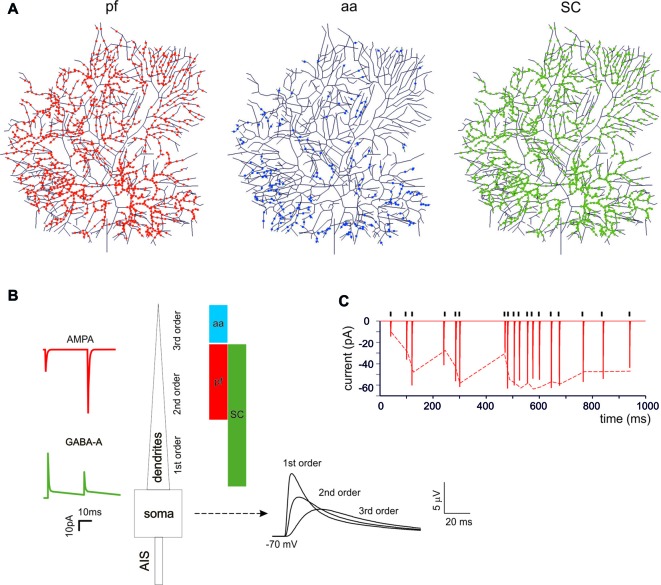
Synaptic activation of the Purkinje cell (PC) neuron model.** (A)** The localization of synapses on PC model dendrites. The dendritic tree receives 1111 excitatory AMPA-type synapses from parallel fibers (pfs; red dots) and 383 from ascending axons (aa; blue dots). The aa are locate on more distal dendritic branches than pf synapses. The GABA-A-type synapses from stellate cells (SCs) were distributed over 1332 dendrites (green dots). **(B)** Unitary EPSCs and IPSCs recorded from the soma. The bars (same colors as in **(A)** indicate the dendritic localization. The EPSPs recorded from the soma show increasing electrotonic filtering moving farther away from soma (Roth and Häusser, 2001). **(C)** Unitary EPSCs elicited in the dendrite using a random stimulation pattern (black ticks) and recorded from the PC model soma. The dotted line represents the experimental result (Dittman et al., 2000).

PCs are endowed with hundred thousand spines (O’Brien and Unwin, 2006), each one receiving a single contact from an aa or a parallel fibers (pf) synapse (Walter et al., 2009). While spines may be critical to implement local biochemical processing during synaptic transmission, they have been suggested to generate linear attenuation with little impact on the overall synaptic excitation process (Ly et al., 2016). Therefore, spines were not reconstructed in the model and excitatory synapses were placed directly on the dendrites. The dendrites were divided into three orders of branching [branch I, composed by 105 sections with diameters between 3.5 μm and 9 μm; branch II, composed by 1111 sections with diameters between 1.2 μm and 3.5 μm; branch III, composed by 383 sections with a diameter between 0 μm and 1.2 μm] receiving specific synaptic inputs. Concerning excitatory synapses (Figure 1A), branch II received only pf synapses and branch III received only aa synapses (Santamaria et al., 2007; Lu et al., 2009; Bower, 2010). Branch I should have received a cf input (Kaneko et al., 2011; Zhang et al., 2015) but this was not used here (not shown). Concerning inhibitory synapses (Figure 1A), these were distributed only on branches I and II for a total of 221 sections, since branches III were experimentally reported not to have inhibitory synapses (Lu et al., 2009; Bower, 2010). Inhibitory synapses on branches I and II were made identical, although those on section I may have a different control (He et al., 2015). The inhibitory BC synapses on the soma and AIS (Iwakura et al., 2012; Blot and Barbour, 2014; Kole et al., 2015) were not used here (not shown).

#### Synaptic Mechanisms

The excitatory and inhibitory synapses were built according to Nieus et al. (2006, 2014) following a modified Tsodyks and Markram formalism (Tsodyks et al., 1998). Model EPSCs and IPSCs were adapted to reproduce unitary synaptic currents recorded from PCs at 37°.

#### The pf/aa—PC Synapses

The glutamatergic AMPA receptor-mediated EPSC model was derived from granule cells and the maximum synaptic conductance was balanced to reproduce a single pf EPSC (Barbour, 1993; Isope and Barbour, 2002). Release probability was adapted to account for pair-pulse facilitation (Zhang et al., 2015). The aa synapses were made with identical physiological properties as the pf synapses (Walter et al., 2009). The AMPA synapse parameters were: release probability = 0.13, τ_REC_ = 35.1 ms, τ_facil_ = 54 ms, τ_I_ = 6 ms, *G*_max_ of 2800 pS, reversal potential = 0 mV. The model as a whole faithfully reproduced the response to random pf stimulations (Dittman et al., 2000). The number of synapses was adjusted to imitate experimental observations of pf bursts that elicited excitatory 250 Hz spike burst in the PC soma (Walter and Khodakhah, 2006).

#### The Stellate Cell (SC)—PC GABA-A Synapses

The gabaergic GABA-A receptor-mediated synaptic mechanism was derived from granule cells and modified by maintaining the alpha1 subunit but deleting the alpha6 subunit (absent in PCs). The value for the fitting were taken from Zhang et al. (2015) to account for *in vivo* recording from stellate cell (SC) in the range P35–P42 to better match the mature PC. The GABA-A synapse parameters were: release probability = 0.35, τ_REC_ = 15 ms, τ_facil_ = 4 ms, τ_I_ = 1 ms and a *G*_max_ = 1200 pS, reversal potential = −60 mV. The number of synapses was adjusted to imitate experimental observations of multiple SC that elicited background inhibitory activity at 1.5 Hz and single SC bursts up to 150 Hz (Zhang et al., 2015).

### Stimulation Protocols

Several stimulation protocols were designed to reproduce the basic patterns used experimentally.

#### Burst Stimulation

This protocol was constructed to investigate the BPR. The fundamental patterns were designed by combining a pf/aa bursts with a SC burst (De Zeeuw et al., 2011; van Beugen et al., 2013; Valera et al., 2016). In a first design, excitatory synapses were randomly distributed over 100 dendrites, both from aa or pf. This allowed to obtain a 250 Hz PC burst as in Walter et al. (2009). The inhibitory burst was composed by three spikes with a 7 ms interspike interval (ISI). Inhibitory synapses were distributed over 25 dendrites and delayed by 4 ms with respect to the excitatory burst to account or synaptic delays along the afferent neuronal chain (granule cell to SC to PC; see also Ramakrishnan et al., 2016). Variants to this pattern were used to reproduce type I, II and III responses (De Zeeuw et al., 2011; Valera et al., 2016), to evaluate the impact of synaptic inhibition, to selectively activate aa rather than pf synapses, to change the intensity or frequency or duration of the bursts, to modify synaptic parameters like release probability and maximum conductance, to restrict pf/aa activity to specific dendritic sectors.

### Data Analysis

The model response was recorded either in voltage clamp or in current-clamp at the soma, and the voltage-dependent ionic conductances were modified in some cases to test their impact on BPR generation. The membrane voltage, the calcium concentration, the ionic currents from each model section were recorded and saved in a nested MATLAB file. The model response properties were analyzed using MATLAB routines (MathWorks, Natick, MA, USA) and custom made Python scripts, using data recorded during each simulations. Each simulation data file contained information about the membrane voltage, ionic channels and synaptic currents recorded in specific location throughout the models.

Biophysical analysis of the model was carried out according to standard theory (Jack et al., 1975). The model electrotonic properties were analyzed using NEURON functions yielding the input impedance, *Z*_in_, for each model section and the corresponding signal attenuation between e.g., a dendritic sections and the soma, *A* = *V*_dend_/*V*_soma_. This allowed to determine the electrotonic distance *L* = ln(A). This definition coincided with the more classical one (*L* = anatomical distance/length constant) defined when a neuron can be reduced through the 3/2 power branching rule to an equivalent cylinder. The NEURON definition proves particularly useful since PCs do not follow the 3/2 power branching rule (Hines and Carnevale, 2001; Hines et al., 2007, 2009).

Since the PC was continuously pacing and input bursts were applied randomly with respect to ongoing spike discharge, repeated PC responses to the inputs differed one from each other. This behavior was represented using raster plots and peri-stimulus time histograms (PSTH), that were constructed using MATLAB routines and were subsequently analyzed to extract the average properties of PC responses.

## Results

The impact of cortical input patterns, conveyed through pf, granule cell aa and stellate cells (SC), on PC spike firing was investigated using detailed PC models differentiated into Z+ and Z− types (Masoli et al., 2015). These models differ for the expression of depolarizing TRPC-like channels in the terminal dendrites, resulting in higher background SS activity in Z− than Z+ PCs (around 90 Hz vs. 45 Hz). Both models were endowed with excitatory and inhibitory synapses, which were located according to anatomical measurements. This, combined with the filtering properties of dendrites, caused the expected EPSP electrotonic decay from synapses to soma (Roth and Häusser, 2001). The synaptic models were endowed with dynamic mechanisms (Tsodyks and Markram, 1997; Nieus et al., 2006, 2014; Figures 1A,B) allowing to adapt neurotransmission to arbitrary spike patterns (Dittman et al., 2000; Figure 1C).

### Burst/Pause Responses (BPRs) in PC Models

**Figure 2:**
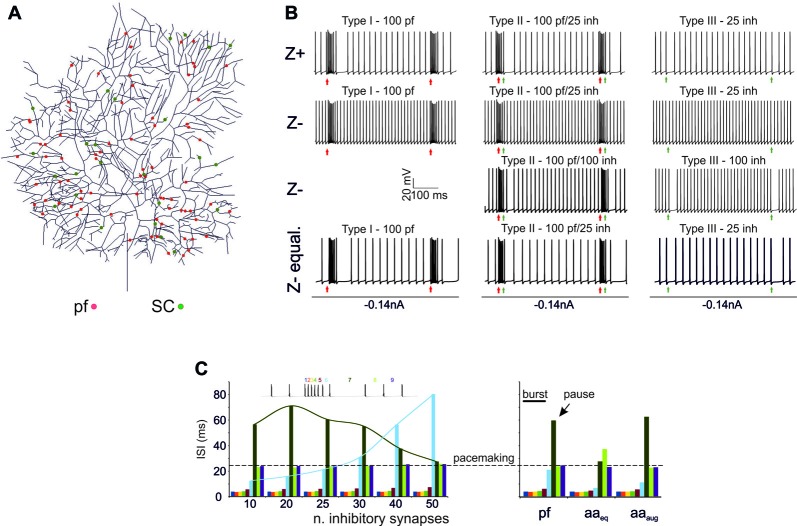
Burst/Pause responses (BPR) in the PC model** (A)** Distribution of the excitatory and inhibitory synapses used for the simulation. The synapses were distributed randomly in the specific dendritic branches. **(B)** Type I, II, III responses in the Z+ and Z− PC models. *Type I* responses were elicited by a 500 Hz—10 spikes burst in 100 pfs. This elicited a ~250 Hz burst, that was followed by a pause in Z+ but not in Z− PC models. *Type II* responses were elicited by a 500 Hz—10 spikes burst in 100 pfs followed by a 130 Hz—three spikes burst in 25 SCs. This elicited a ~250 Hz burst, that was followed by a pause in Z+ but not in Z− PC models. The pause occurred in the Z− model only when the number of SC synapses was increased to 100. *Type III* responses were elicited by a 130 Hz—three spikes burst in either 25 SCs. This elicited a pause in Z+ but not in Z− PC models. The pause occurred in the Z− model only when the number of SC synapses was increased from 25 to 100. The Z− behaved similarly to Z+ model when the Z− was equalized to Z+ basal firing rate with somatic injection of a constant negative current.** (C)** *Left*, Sensitivity of the burst to the number of inhibitory synapses. The interspike interval (ISI) for each spike is reported. Increasing the number of inhibitory synapses does not change the burst but generates progressively longer pauses with a sharper burst/pause transition. *Right*, using the example with 25 SC synapses for comparison, the impact of ascending axons (aa) stimulation is evaluated. When aa are made identical to pf synapses, there is no clear pause generation. However, this occurs when the aa synapses are potentiated by increasing their postsynaptic maximum conductance and presynaptic release probability (Sims and Hartell, 2005, 2006) demonstrating “functional equivalence” with pf synapses (Walter et al., 2009) in BPR.

A first question was whether the PC model was able to generate BPRs following activation of excitatory and inhibitory synapses (Cao et al., 2012; Herzfeld et al., 2015) and whether BPRs were different between Z+ and Z− PCs. The PC responses to input bursts generated by Granule cells (Rancz et al., 2007; van Beugen et al., 2013; Powell et al., 2015; Wilms and Häusser, 2015; Delvendahl and Hallermann, 2016) were tested in Z+ and Z− PC models in three stereotyped functional cases corresponding to the definitions of type I, type II and type III responses reported *in vivo* (De Zeeuw et al., 2011). Type I responses were driven by pure Granule cell excitatory inputs (100 synapses), type II responses were driven by both granule cell excitatory inputs (100 synapses) and SCs inhibitory inputs (25 synapses), type III responses were driven by SC inhibitory inputs only (25 synapses; Figure 2). In all cases, molecular layer interneuron inhibition was delayed by 4 ms to account for delays accumulated along the synaptic chain (Eccles et al., 1967; Ramakrishnan et al., 2016). In this set of simulations, a random distribution of active synapses was used and maintained the same with all the different stimulation patterns (Figure 2A).

#### *Z*+ Model (Figure 2B)

In Type I responses, a brief pf burst elicited a PC burst terminating in close coincidence with the stimuli (average frequency of 260 Hz) followed by a pause, configuring a typical BPR. The pause between the last spike of the burst and the first spikes when SS activity restarted showed an ISI of 50.46 ± 25.6 ms. Type II responses showed a similar BPR as type I, with the pause showing an average ISI of 48.2 ± 20.4 ms (Steuber et al., 2007). Type III responses showed just the pause, with an ISI of 27.4 ± 3.7 ms. Thus, with this stimulation pattern, BPRs were determined by intrinsic properties of Z+ PCs and were accentuated by molecular layer interneurons.

#### *Z*− Model (Figure 2B)

In the Z− model, a 10-pulses/500 Hz pf burst generated a PC burst like in the Z+ model type I and type II responses. However, the Z− model was almost unable to generate any pauses, in either type I, II or III responses. This difference with the Z+ model was reduced by raising synaptic inhibition from 25 to 100 molecular layer interneuron synapses, which allowed pauses to emerge in the type I and type II responses (see Figure 2C). Therefore, the Z− PC model showed reduced ability to generate intrinsic BPR, in which the pauses were markedly dependent on the amount of molecular layer interneuron inhibition. It should be noted that the reduced ability of Z− PC models to generate intrinsic BPRs was likely to be related to the high basal firing rate, since Z− became similar to Z+ BPR when the basal firing rate was equalized with somatic injection of a constant negative current (Figure 2B).

Modulation of BPR was investigated in detail in Z+ PC type II responses, in which BPR was the most pronounced. Increasing the strength of inhibition did not change the burst but prolonged the pause. Moreover, responses to aa synapses were differentiated by considering their specific location on distal dendrites. The aa generated weaker BPR than pf probably because of their longer electrotonic distance from soma (see also Figure 6A). However, the aa and pf BPR became very similar when aa transmission strength was increase, reproducing the functional equivalence of transmission along these two transmission lines (Sims and Hartell, 2005, 2006; Walter et al., 2009).

**Figure 3:**
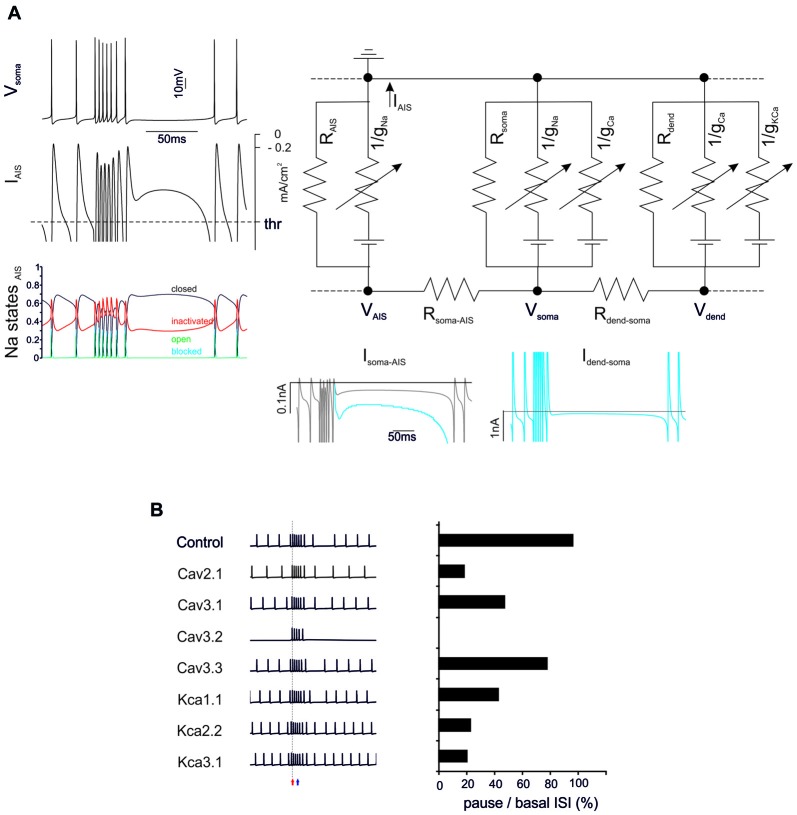
General mechanisms of BPR. **(A)** The electrical circuit provides a schematic representation of the compartmentalization of the PC model, highlighting the axon initial segment (AIS), soma and dendrites. Membrane potential in the main nodes (*V*_AIS_, *V*_soma_, *V*_dend_) depends on current flow along the internal resistances (*R*_soma-AIS_, *R*_dend-soma_) and through the membrane of each compartment (including leakage resistances and voltage-dependent ionic channels). Traces on the *left* show the BPR (enhanced for the purpose of clarity by increasing KCa by 50%) along with the current flowing through the AIS membrane (the action potential current threshold is indicated) and the AIS Na channel states (open, closed, inactivated and blocked). Traces on the *bottom* show the current flowing through internal resistances from dendrites to soma and AIS. The action potential current threshold is indicated by dotted lines (see “Materials and Methods” Section for details). **(B)** Effect of ionic channel knock-out from the PC model on BPR. *Left*, voltage traces. *Right*, corresponding percent change of the ratio between pause and basal ISI in the different conditions.

**Figure 4:**
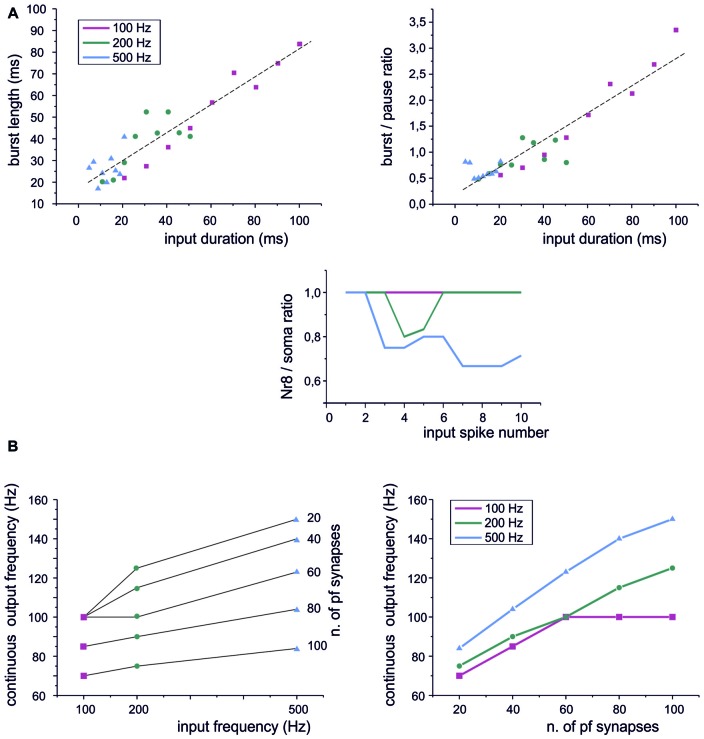
Sensitivity of PC responses to differences in input spike patterns.** (A)** Type I BPRs were elicited using different input frequencies and burst durations. *Left*, the relationship between output bursts length and the duration of input burst at 100 Hz, 200 Hz, 500 Hz. A linear fitting is superimposed (*R*^2^ = 0.996, *p*_(F)_ < 10^−6^). *Right*, the relationship between the burst/pause ratio (duration of burst by duration of pause) and the duration of input burst at 100 Hz, 200 Hz, 500 Hz. A linear fitting is superimposed (*R*^2^ = 0.96, *p*_(F)_ < 10^−4^). *Bottom*, PC burst transmission across the 8^th^ Ranvier node for input burst at 100 Hz, 200 Hz, 500 Hz. **(B)** Continuous spike frequency modulation was elicited using input spike trains with different frequencies and number of pf synapses.

**Figure 5:**
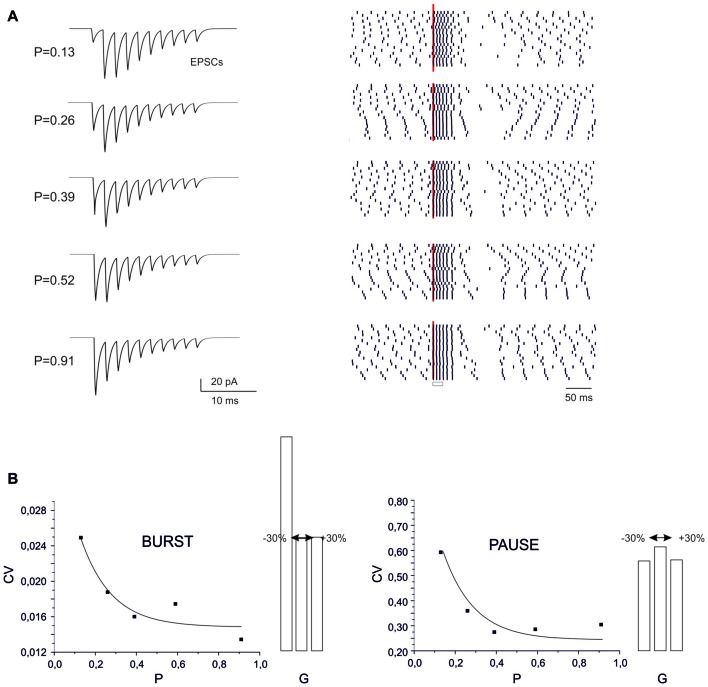
Sensitivity of BPR to synaptic parameters. ** (A)** Type II BPRs were elicited by 500 Hz-10 spikes bursts using different release probabilities, *p*. By increasing *p*, the EPSCs trains changed progressively from facilitation to depression. The raster plots obtained over repeated trails reveal an increased precision of spike emission by increasing release probability, but this cannot be detected in the peri-stimulus time histograms (PSTH). **(B)** The CV of the ISI measured from raster plots shows a decrease both for the burst and pause by increasing *p*. Decaying exponential fitting is superimposed (bursts, *R*^2^ = 0.98, *p*_(F)_ < 10^−6^; pause, *R*^2^ = 0.996, *p*_(F)_ < 10^−6^). The histograms show the changes caused by a ±30% change in AMPA maximum conductance.

**Figure 6:**
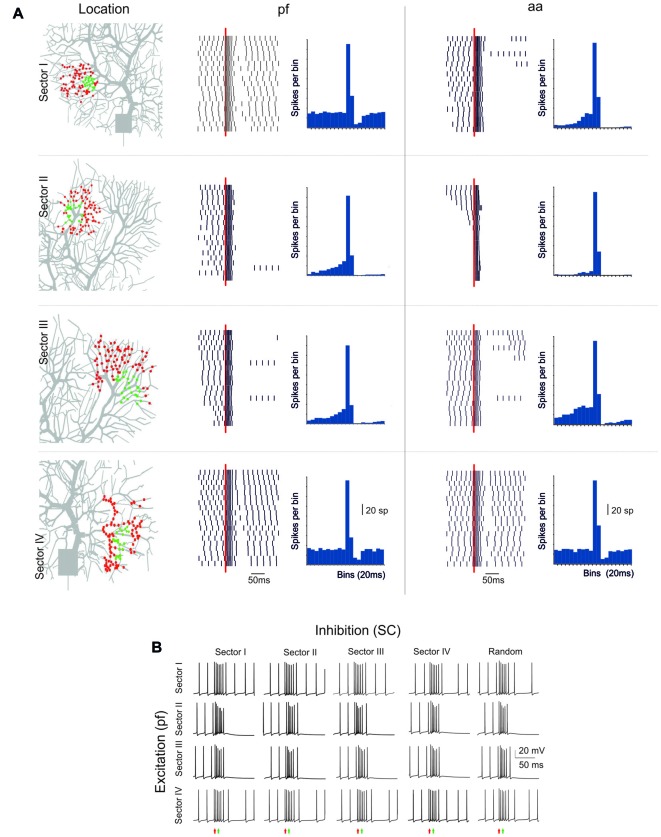
Sensitivity of BPR to dendritic localization.** (A)** Type II BPRs were elicited by 500 Hz—10 spikes pf or aa bursts in four different dendritic sectors, labeled I to IV. SC synapses were activated by 130 Hz—three spikes trains and were located in the same sectors. In all cases the number of pf or aa and SC synapses was the same as in the case of random synapses distribution. *Left*, distribution of active pf synapses. The raster plots and PSTH reveal that BPRs are different in the four sectors, mostly due to a different duration of the pause. *Right*, effect of activation of aa synapses in the same sectors and with the same inhibitory synapses. Note that the raster plots and PSTH look similar to those of pf except for sector I, in which the pause is longer. **(B)** Different localization of inhibitory synapses has a marginal effect on BPRs elicited by pf synapse (almost the same happened for aa synapses, not shown).

### The Ionic Basis of BPR

The BPRs mechanism was analyzed by tracking transmembrane currents and intracellular axial currents in different cellular compartments using the Z+ PC type I model, in which the intrinsic ability to generate BPRs was evident (Figure 3A). In the PC model, APs arise from the AIS (Masoli et al., 2015) and, not surprisingly, the pause is characterized by a protracted decrease of the AIS transmembrane current (*I*_AIS_; Jack et al., 1975). In fact, this makes the pause appearing as an interruption of pacemaking. Pacemaking in PCs is sustained by the persistent Na currents generated mostly in AIS and by Ca currents generated mostly in the dendrites and transmitted to the AIS through the internal resistance (Llinás and Sugimori, 1980a,b), providing two candidate mechanisms for pause generation.

Na currents in the AIS may be inactivated during the burst and then take time to recover, reducing the depolarizing drive. However, there was no remarkable inactivation of Na currents during the bursts that might prevent reactivation of the pacemaker. Therefore, this first mechanism could be excluded.

Ca channel activation and the consequent Ca entry into the dendrites may activate KCa currents overtaking inward currents and generating a protracted repolarizing drive. Actually, the current transmitted from the dendrite to soma (*I*_dend-soma_) and from soma to AIS (*I*_soma-AIS_) was reduced during the pause compared to pacemaking regime. Therefore, reduced depolarizing current transmission from the somato-dendritic compartment to AIS appears as the main responsible of the pause.

The ionic nature of the mechanism was confirmed by specific manipulations of the Ca/KCa ionic mechanisms. When either dendritic KCa conductances or Ca conductances were set to zero, the BPR was altered with a marginal reduction of burst duration (from 0% to −16.7%) but a much more dramatic reduction of the pause (from −20% to −80%) (Figure 3B). It should be noted that all Ca channels proved critical for BPR, especially Cav2.1 and Cav3.1 (Cav3.2 switch-off blocked pacemaking; Masoli et al., 2015) as well as all KCa channels, including KCa1.1, KCa2.2, KCa3.1.

These observations show that making BPRs is an intrinsic property of the PC model, which depends on the generation of large KCa currents in the dendrites. In Z− PCs, the mechanism were the same as in Z+ PCs except that TRP channels injected a constant inward current through the dendrites counterbalancing KCa and making the pause more difficult to elicit (not shown).

### Dependence of BPR on Input Spike Trains

A central issue in cerebellar physiology is how PCs transform signals coming from granule cells into specific outputs. The spike patterns emitted by granule cells in response to punctuate stimulation consist of short bursts composed of spikes with variable number and ISI (Chadderton et al., 2004; Rancz et al., 2007; D’Angelo and De Zeeuw, 2009). We have therefore used the PC model to simulate the impact on BPR of stereotyped patterns composed by a fixed number of pulses at different frequencies. Concerning burst length, the PC model showed an almost linear input/output relationship at frequencies ranging from 100 Hz to 500 Hz (Figure 4A, left). The pause also showed a slight dependency on the length of the input burst and this eventually caused the burst/pause ratio to reliably report the duration of the input burst (Figure 4A, right). There are two additional noteworthy properties. First, the linearity of burst and burst/pause coding was maintained almost independently from the input frequency. Secondly, the Ranvier nodes in the PC axon filtered the highest frequencies (Masoli et al., 2015) so that the burst was more reliably transmitted at the lower input frequencies (Figure 4A, bottom).

Another modality of granule cell response, largely investigated in the vestibulo-cerebellum, is to generate spike trains in response to prolonged mossy fiber inputs (Arenz et al., 2008). When such stimuli were used, the PC model followed the input frequency (Figure 4B, left), although the frequency dependence was rather weak (as also noted for the BPR in Figure 4A). However, there was a steep relationship between the output frequency and the number of active synapses (Figure 4B, right). Therefore, the PC model was more efficient in detecting the intensity of the granule cell input rather than the frequency at individual synapses.

### Dependence of Burst-Pause Precision on Synaptic Parameters

The recoding of input into output spike patterns in PCs is thought to depend on how long-term synaptic plasticity modifies PC synaptic responsiveness. While classical pf-PC LTD has postsynaptic expression and simply causes a scale-down of postsynaptic currents, a presynaptic change in release probability (*P*) would modify neurotransmission dynamics (Tsodyks and Markram, 1997; Nieus et al., 2006). These can shift from the typical short-term facilitation at low *P* to short-term depression at high *P*. These changes can therefore differentially affect the PC BPR.

At different *P* values (range 0.1 and 0.9), the overall response pattern in PC output bursts did not change remarkably. In all cases, the output spikes followed the input spikes quite closely during the burst, then one or two extra spikes were generated and the pause occurred. However, at a closer inspection of raster plots, the precision of action potential emission changed. By increasing *P* (from *P* = 0.13 to *P* = 0.91) the PC model responses were characterized by a greater precision, with decreased variability in burst spike pattern and pause length (see also Figure 2C). Increased precision derived from the decreased paired pulse ratio (A2/A1, where A1 and A2 are the amplitudes of the first and second response in a pair). In this way, the first EPSP in the train became more precisely aligned with the input burst leading to a repeatable spike generation independent from the relative phase of background firing activity (see also Figure 8 below). Therefore, the highest precision was obtained when the pf-PC synapse expressed presynaptic pf-PC LTP. This suggests that release probability controls fine regulation of spike timing on millisecond scale.

**Figure 7:**
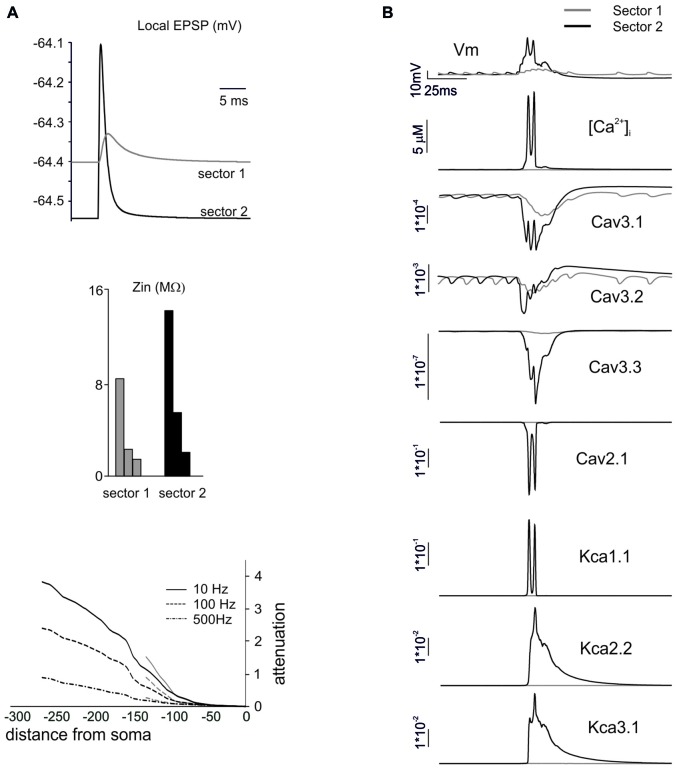
The mechanisms differentiating BPRs among dendritic sectors. In these simulations on a Z+ PC model, type II BPRs were elicited by 500 Hz—10 spikes pf bursts in dendritic sectors I and II and SC synapses were activated by 130 Hz—three spikes trains in the corresponding pf sectors (see Figure 6A). **(A)** Local generation of minimal EPSPs in one of the terminal compartments of dendritic sectors I (~140 μm from soma) and II (~270 μm from soma). Note the larger and faster EPSP in sector II than I. In the same dendritic compartments, Zin is about twice as large in sector II than I at 10, 100 and 500 Hz. Correspondingly, attenuation follows different profiles in sector I and II. **(B)** Local currents in one of the terminal compartments of sectors I and II. Note that, in sector 2, the EPSPs trigger a local Ca spike along with a strong activation of Ca currents (Cav3.1, Cav3.2, Cav3.3, Cav2.1) and KCa currents (KCa1.1, KCa 2.2, KCa3.1) and a large [Ca^2+^]_i_ increase. In sector I, the depolarization is too weak to activate the Ca spike so that ionic currents and [Ca^2+^]_i_ changes are correspondingly much smaller.

**Figure 8:**
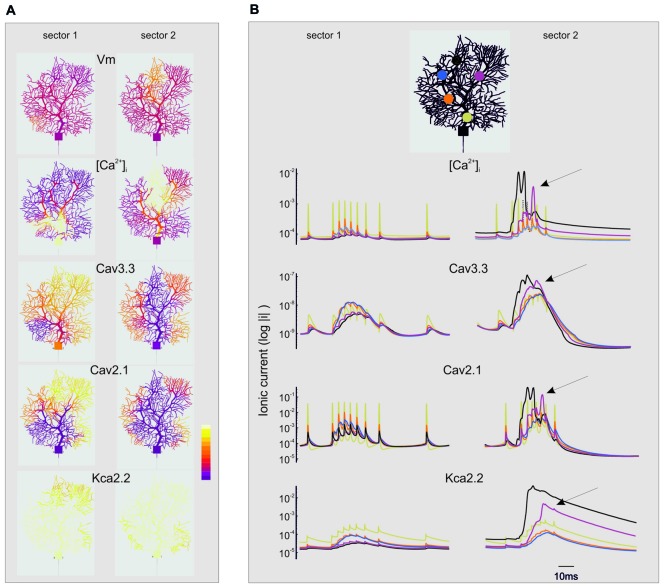
Spatio-temporal evolution of BPRs in the dendrites. The figure extends the description of the simulations shown in Figures 6, 7. For simplicity, only the currents showing the largest changes are shown (Cav3.3, Cav2.1, KCa 2.2) along with [Ca^2+^]_i_. Note the log scale (all parameters are made positive) to account for large variations on the ordinate. **(A)** Spatial profile of ionic currents and [Ca^2+^]_i_ changes. Note the larger extension of dendritic activation when stimuli are delivered to sector II than I. Synaptic stimuli are given either in sector I and II. The color scale shows relative values for Vm (−80; 40 mV), [Ca^2+^]_i_ (4.5*10^−8^; 3*10^−7^ M/l), I_Cav3.3_ (−1*10^−9^; 0 mA/cm^2^), I_Cav2.1_ (−0.001; 0 mA/cm^2^), I_KCav2.2_ (0; 1*10^−5^ mA/cm^2^). **(B)** Temporal profile of ionic currents and [Ca^2+^]_i_ changes taken from five different compartments indicated by colors (see the legend on top). Although the synaptic stimulus is given only in sector I or II, there is a progressive invasion of neighboring dendritic regions that can lead to full-blown Ca spikes and large ionic current activation outside the stimulated sector (e.g., arrows). These dynamic changes are more marked when stimuli are delivered to sector II than I.

In the case of a postsynaptic change in synaptic conductance *G* (−30% to 30% with respect to control), the only remarkable effect was a reduction in burst spikes precision when *G* was reduced, while no appreciable changes were observe in pause precision. The spike precision decrease at low *G* was likely related to a greater influence of previous spikes on burst initiation. Therefore, in the present conditions, precision could be more effectively tuned by *P* than *G*.

### The Influence of Dendritic Sectors on PC Response Patterns

The PC shows complex branching (Nedelescu and Abdelhack, 2013) and active electroresponsiveness in the dendrites, so that the specific location of afferent synapses may influence the response pattern. The model was exploited to redirect to specific dendritic sectors (numbered I–IV) the same stimuli that were used before for synapses randomly distributed over the whole dendritic tree (see Figures 1–6).

The PC response was different depending on the stimulated sector (Figure 6A). When excitatory pf and inhibitory stimulations were delivered to dendritic sector I or IV, the PC BPRs were similar to those obtained using a random distribution of synapses. However, when excitatory stimulation was delivered to sector II or III, the pause was much longer than usual (over 250 ms in the Z+ model). When aa was substituted to pf stimulation, the responses were similar except for sector I, which showed a longer pause with aa then pf stimulation (the reason of this will be explained below). The difference between sectors was poorly sensitive to the location of inhibition, and BPRs did not change remarkably when inhibition was moved to sectors different from the one that was excited by pf synapses (Figure 6B). Similar response properties were observed in the Z− model, although pauses were shorter (data not shown).

In order to understand the mechanism differentiating responses among dendritic sectors, we compared sector I to sector II, which showed a remarkably different pause lengths. The impact of dendritic structure was considered first (Figure 7A). The local currents generated by synaptic activation (that were identical in the two sectors) caused a stronger depolarization in sector II than sector I. This reflected the different *Z*_in_, that was about twice as large for sector II than sector I. Accordingly, this determined different voltage attenuation profiles, so that sector I depolarization started from a higher level and then decayed over a longer distance while approaching the soma. It should be noted that, in these simulations, we used single-synapse stimuli causing small depolarizations from a hyperpolarized membrane potential, so that voltage-dependent currents were not remarkably activated.

Then we considered the activation of voltage-dependent currents in the dendrites (Anwar et al., 2014) by delivering the appropriate multisynaptic stimulation pattern to the PC model in pacemaking regime. The higher synaptic depolarization in sector II than in sector I resulted in a stronger voltage-dependent activation of Ca channels in the former than in the latter (Figure 7B). In sector II, LVA currents amplified the EPSPs leading membrane potential to raise enough to activate also the HVA currents, thus causing a regenerative calcium spike. Consequently, a large raise in intracellular calcium activated the KCa system. This effect eventually influenced the spike-generating mechanisms in the AIS and regulated the pause, that was longer in sector II than sector I.

### Threshold and Spread Effects of Dendritic Activity

The non-linear nature of voltage-dependent mechanisms of BPR and their topographical nature bear about two main consequences.

#### “Spread” Effect (Figure 8A)

While sector I phenomena remained almost locally confined, the voltage-dependent effects initiated in sector II rapidly spread to neighboring sectors and eventually to the whole dendrite (Figure 8B). Full-blown calcium spikes appeared with some delay in a region close to sector II, involving LVA followed by HVA calcium channel activation causing a large [Ca^2+^]_i_ raise and eventually KCa activation and the pause. Therefore, also dendritic sectors that are not activated synaptically can generate a local [Ca^2+^]_i_ increase and take part to control the BPR pause.

#### “Threshold” Effect

**Figure 9:**
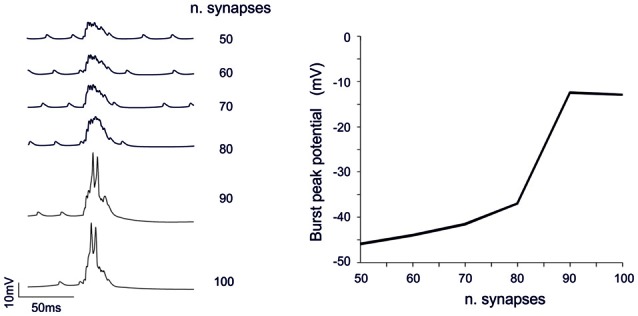
Threshold effects in dendritic sectors. In these simulations on the Z+ PC model, type II BPRs were elicited by 500 Hz—10 spikes pf bursts in dendritic sectors II and SC synapses were activated by 130 Hz—three spikes trains in the same sector. The number of active pf synapse was increased from 50 to 100. Traces on the left show membrane potential in a compartment of sector II. The dendritic response remained sub-threshold until around 70 synapses and then jumped steeply over-threshold generating Ca spike at 80 synapses. The plot on the right shows the amplitude of the dendritic depolarization as a function of the number of activated synapses.

The analysis of Figures 7, 8 shows that voltage-dependent channels in sector II (but not sector I) cross the activation threshold, so that a local Ca spike is activated bringing about a remarkable KCa channels activation and a long pause. In sector II, Ca spikes and threshold crossing could be prevented by down-tuning the synaptic input. Indeed, when the number of pf synapses was increased progressively from 50 to 100, a sharp threshold in Ca spike activation was observed around 70–80 synapses. Interestingly, with sub-threshold responses the BPR of sector II became almost the same as for sector I (Figure 9).

### Spatio-Temporal Integration in the PC Dendrite

A last question is how responses generated in different dendritic sectors can integrate to generate BPRs.

#### Spatial Integration

**Figure 10:**
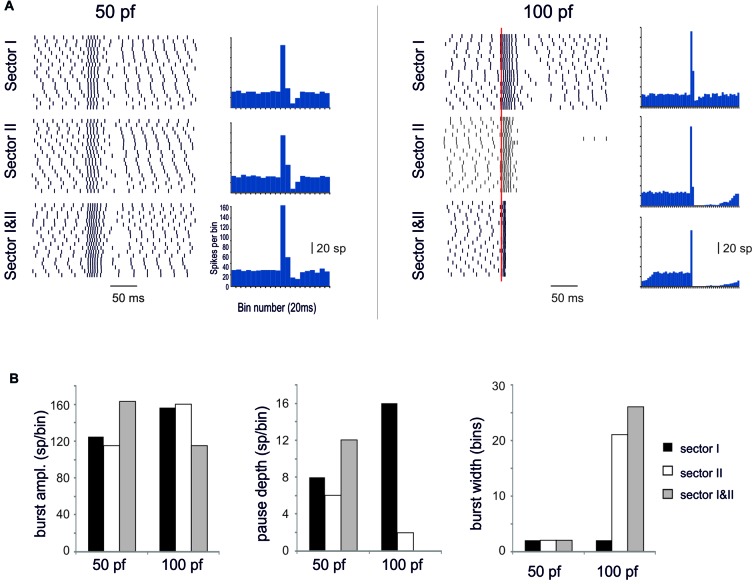
Spatial integration in the dendrites. In these simulations on the Z+ PC model, type II BPRs were elicited by 500 Hz—10 spikes pf bursts in dendritic sectors I and II and SC synapses were activated by 130 Hz—three spikes trains in the same sectors. The number of active pf synapse was either 50 or 100. **(A)** The raster plots and PSTH show model responses when sectors I and II are activate alone or together. **(B)** The histograms show the amplitude of PSTH peak and the depth and duration of the pause in the different cases.

When sector I and II were activated together, different behaviors appeared depending on the intensity of dendritic activation. Below Ca spike threshold, the conjoint BPR showed a burst enhancement but the pause was almost the same as in the two sectors alone (Figure 10). Conversely, when sector II Ca spike was supra-threshold, BPRs were dominated by the pause (Figure 10). The BPR burst became shorter and smaller and the pause longer and deeper than in any one of the two sectors activated alone. This was likely to reflect boosting of KCa current activation due to the conjoint action of the two sectors.

#### Temporal Integration

**Figure 11:**
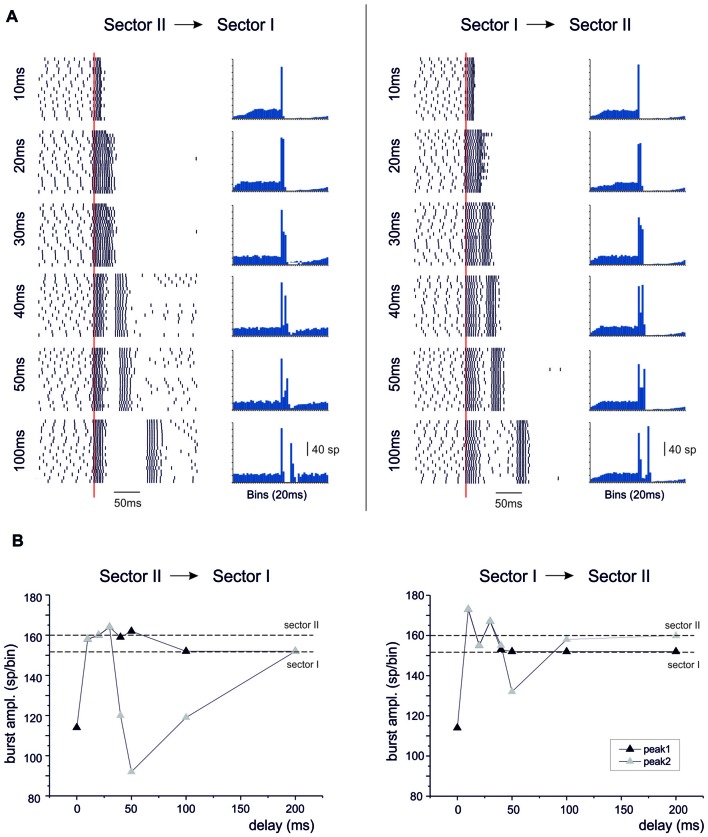
Temporal integration in the dendrites. In these simulations on the Z+ PC model, type II BPRs were elicited by 500 Hz—10 spikes bursts to 100 pfs in dendritic sectors I and II and SC synapses were activated by 130 Hz—three spikes trains in the same sectors. Following activation of one sector, the other was delayed by 10–200 ms. **(A)** The raster plots and PSTH show model responses when sectors II precedes sector I (*left*) or when sectors I precedes sector II (*right*). **(B)** The plots show the amplitude of PSTH peaks in the different cases. The levels of isolated sector I and II responses are indicated by dashed lines.

When activation in sector I preceded sector II, or vice versa, the bursts were initially fused together but then separated generating characteristic response profiles (Figure 11). When sector II preceded sector I, the unified burst initially decreased and then approached the level of the burst in sector II before separating into two individual bursts. For longer delays, the second burst was reduced along the time course of the BPR pause and finally recovered to sector I burst amplitude. When sector I preceded sector II, the responses behaved similarly. Starting from a reduced conjoint burst, the burst amplitude increased toward that of the burst in sector I. For longer delays, the second burst was reduced along the time course of the BPR pause and finally recovered to sector II burst amplitude.

## Discussion

The present simulations allowed to investigate the foundations of PC responsiveness to synaptic bursts in a way that would not be possible with experiments only. Using the model, we could predetermine the composition of synaptic input patterns and analyze the neuron response mechanisms by independently monitoring several parameters (like membrane potential, ionic currents and calcium concentration) over multiple dendritic compartments. In response to pf and SC inputs, the PC model generated BPRs based on voltage-dependent activation of the dendritic Ca-KCa channel system. BPRs could reliably represent complex granular and molecular layer input patterns and depended on the specific sector of the dendritic tree that was stimulated, on the ionic channel complement, and on the excitatory/inhibitory synaptic pattern. These simulations suggest therefore that PCs exploit their intrinsic electroresponsiveness and the input pattern topography on the dendrites in order to generate a flexible BPR code (Herzfeld et al., 2015) to be relayed to DCN neurons.

### Calibration and Validation of PC Model Synaptic Responsiveness

This investigation was made possible by the use of an advanced model of the PC (Masoli et al., 2015), which was developed from earlier ones (De Schutter and Bower, 1994a,b; Rapp et al., 1994; Roth and Häusser, 2001; Steuber et al., 2007; Masoli et al., 2015) by including novel electrophysiological features and was validated against a large set of recent experimental data. The model was implemented with ionotropic synapses coming from granule cells (both aa and parallel fibers) and SCs. Additional synaptic mechanisms that may affect the response to specific input patterns (e.g., see Barbour et al., 1994; Takahashi et al., 1995; Tabata et al., 2005; Blot and Barbour, 2014) remain to be assessed.

The pf synaptic transmission was calibrated to match responses evoked by random input patterns (Dittman et al., 2000), ensuring that pf synapses could precisely reproduce the temporal dynamics of short-term synaptic plasticity. The aa were made identical to pf synapses but were located on distal rather than proximal dendrites. A potentiation of the aa (Sims and Hartell, 2005, 2006; Walter et al., 2009) was required to counterbalance the longer electronic distance (*L* = 1.4 vs. *L* = 0.4) from soma (Roth and Häusser, 2001), thus ensuring the reported functional equivalence of aa and pf synapses (Walter et al., 2009).

It should be noted that in no case the model generated bistable switching between up-down states (Loewenstein et al., 2005), which may require additional mechanisms or specific modulation of receptors and ionic channels.

### Model Predictions on BPR Coding Properties

Model simulations brought about a number of testable predictions.

1. The *bursts* in type I and type II PC responses (De Zeeuw et al., 2011) was similar, in agreement with the fact that inhibition arrives after the burst is terminated (typically 5–10 ms later, Ramakrishnan et al., 2016). Therefore, the granule cell output patterns could directly reflect onto the PC burst, supporting experimental observations (Cao et al., 2012; Herzfeld et al., 2015).
2. In the Z+ PC model, the *pause* was largely determined by intrinsic membrane properties and was accentuated by molecular layer interneuron inhibition, while in the Z− PC model, the pause was much more strictly dependent on SC inhibition. It is therefore possible that PCs in Z+ and Z− zones undergo different control schemes through the inhibitory interneuron network of the molecular layer.
3. In response to random synapse stimulation, the *burst*: (i) started precisely in coincidence with the arrival of synaptic inputs; (ii) had a duration faithfully reproducing that of synaptic inputs; (iii) had a frequency proportional to input intensity; but (iv) was poorly sensitive to input frequency. Moreover, (iv) the burst and pause length co-varied generating a linear relationship with the input burst length. Therefore, the timing and duration of granule cell discharge and the number of discharging granule cells would be the most critical parameters controlling the burst (Arleo et al., 2010; Galliano et al., 2013).
4. *Burst* and *pause* precision could be fine-tuned on the millisecond time-scale by pf release probability but much less so by receptor conductance, suggesting a specific role for presynaptic forms of plasticity at the pf-PC synapse in tuning transmission precision (Hansel et al., 2001; Gao et al., 2012).
5. The BPR demonstrated a remarkable sensitivity to synapse location that caused the modulation of *pause* length. Therefore, the geometrical arrangement of input patterns could be important to shape BPR (Bower, 2010; Abrams and Zhang, 2011; Wilms and Häusser, 2015; Valera et al., 2016), e.g., by differentiating responses to aa and pf synapses (Bower, 2002) or by exploiting prewired connections from granule cell clusters (Valera et al., 2016).
6. Different dendritic sectors could interfere one with each other in determining complex BPR combinations and sequences (Santamaria and Bower, 2005). The interference was determined by the generation of non-linear supra-threshold events driven by Ca spikes. Threshold crossing depended on a minimal number of active synapses and was enhanced by the high input conductance of terminal regions. As an important consequence, this would make the aa superior to pf synapses for generating dendritic Ca spikes and in controlling dendritic response patterns (Bower, 2002).

These observations suggest that BPRs represents a flexible coding strategy accounting for the timing, duration and intensity of input granule cell spike patterns and that can be modulated by the spatiotemporal distribution of the input, by its intensity, by synaptic plasticity and by inhibitory interneuron activity.

### Model Predictions on Dendritic Processing

Model simulations also helped hypothesizing how PC dendrites process incoming synaptic inputs through BPRs. Dendritic Ca channels, primed by pf or aa EPSPs, would generate a depolarizing current flowing through the dendrites and activate a spike burst in the AIS. The corresponding raise in [Ca]_i_ would activate KCa channels causing a protracted hyperpolarization, interrupting pacemaking and generating the pause. The whole process turned out to be very sensitive to the location of synaptic inputs on the dendrites. Actually, EPSPs amplitude was larger at longer electrotonic distances and this enhanced the *threshold* crossing for generating local Ca spikes, that could then *spread* around invading neighboring dendritic regions. Thus, the combined effect of dendritic structure and voltage-dependent ionic channels generated non-linear interactions sculpting the spatio-temporal profile of local [Ca]_i_ and BPRs. It is tempting to speculate that this would eventually extend the plastic and computational properties of PCs beyond the *linear perceptron* hypothesized previously (Brunel et al., 2004).

A peculiarity of the PC dendrites is the enrichment in Ca channels (Llinás and Sugimori, 1980a,b), which include both HVA (Cav2.1) and multiple LVA (Cav3.1, Cav3.2, Cav3.3) subtypes (Ly et al., 2016). While Cav2.1 (the classical PC P-type channel) was known to regulate KCa1.1 (BK) channel activation and therefore the fast action potential AHP, the role of LVA channels remained uncertain. The present simulations show that LVA Ca channels (including at least cav3.1 and Cav3.3) are critical for BPR, since they are activated during the burst and then play a local but key role in activating the KCa2.2 (SK2) and KCa3.1 (SK4) channels, thereby regulating the pause. The relevant role of LVA calcium channels in EPSP generation by input bursts has recently been reported experimentally (Ly et al., 2016).

It should be noted that, when simulating intrinsic electroresponsiveness with somatic recordings, LVA Ca channels and KCa channels proved either noncritical or subcritical (Masoli et al., 2015). Therefore, in light of the present simulations, the tuning procedures of maximum ionic conductances in neuronal models should be targeted toward features addressing synaptic response pattern (Marasco et al., 2013) rather than just responses generated by somatic current injection (Druckmann et al., 2007, 2008; Masoli et al., 2015, 2017). Since some of these channels are located in the spines (specifically Cav3.1), a future assessment of the impact of spines in the PC model is warranted.

## Conclusions: PC Coding Properties and Testable Model Predictions

In aggregate, these simulations showed that BPRs could fully exploit dendritic ionic channels (Llinás and Sugimori, 1980a,b) to generate voltage-dependent responses. However, this mechanism did not compromise the linearity of BPR input-output relationships supporting the predictions that PC may work as a linear perceptron (Brunel et al., 2004) and a perfect integrator (Phoka et al., 2010). Simulations supported the concept that BPRs represent a fundamental operating mode of PCs (Walter and Khodakhah, 2006, 2009; Chen et al., 2016). First, BPRs could discriminate among different input patterns coming from the mossy fibers and expanded/recoded in the granular and molecular layer (Marr, 1969). Second, BPRs sensitivity on the millisecond scale would allow PCs to operate as precise temporal devices (Eccles, 1973). Third, BPRs could discriminate among synaptic locations, generating an exquisite sensitivity to the topography of input patterns (Migliore et al., 2008). Finally, BPRs could generate complex combinations and sequences (Santamaria and Bower, 2005). By expressing these properties, BPRs would effectively integrate the spatio-temporal activity patterns generated in the cerebellar cortex into salient engrams, a prediction warranted experimental testing through electrophysiological, imaging and optogenetic recordings.

## Author Contributions

SM wrote the model, carried out the simulation and took part to manuscript writing and discussion. ED coordinated the work and wrote the manuscript.

## Conflict of Interest Statement

The authors declare that the research was conducted in the absence of any commercial or financial relationships that could be construed as a potential conflict of interest.
